# Reciprocal Regulation of GLI1 and GLI3 Fine Tunes the Pathogenic Behavior of Synovial Fibroblasts in Rheumatoid Arthritis

**DOI:** 10.1111/1756-185x.70690

**Published:** 2026-06-05

**Authors:** Motohiko Sato, Tetsuya Saito, Yoji Komiya, Seiji Noda, Yasuhiro Tagawa, Akio Yamamoto, Hideyuki Iwai, Kentaro Endo, Hideyuki Koga, Yasuhiro Takahara, Kazutaka Sugimoto, Ichiro Sekiya, Eiryo Kawakami, Tadashi Hosoya, Shinsuke Yasuda

**Affiliations:** ^1^ Department of Rheumatology, Graduate School of Medical and Dental Sciences Institute of Science Tokyo (IST) Tokyo Japan; ^2^ Center for Stem Cell and Regenerative Medicine Institute of Science Tokyo (IST) Tokyo Japan; ^3^ Department of Joint Surgery and Sports Medicine, Graduate School of Medical and Dental Sciences Institute of Science Tokyo (IST) Tokyo Japan; ^4^ Department of Orthopedic Surgery Nippon Kokan Fukuyama Hospital Hiroshima Japan; ^5^ Department of Orthopedics Sonodakai Joint Replacement Center Hospital Tokyo Japan; ^6^ Department of Artificial Intelligence Medicine, Graduate School of Medicine Chiba University Chiba Japan; ^7^ Advanced Data Science Project (ADSP) RIKEN Information R&D and Strategy Headquarters Kanagawa Japan; ^8^ NEXT‐Ganken Program Japanese Foundation for Cancer Research (JFCR) Tokyo Japan; ^9^ Institute for Advanced Academic Research (IAAR) Chiba University Chiba Japan

**Keywords:** fibroblast, GLI1, GLI3, hedgehog signaling pathway, rheumatoid arthritis

## Abstract

**Objective:**

We investigated the role of GLI3, a transcription factor highly expressed in the pathogenic THY1^+^CD34^−^ sublining subset of rheumatoid arthritis synovial fibroblasts (RASFs), in regulating their pathogenic behavior.

**Methods:**

GLI3 protein levels were quantified in freshly isolated RASF subsets by Western blotting. Bulk RASFs were subjected to siRNA‐mediated knockdown (KD) of GLI3 or GLI1, followed by RNA sequencing. The effects of GANT61 on RASF proliferation, cell‐cycle progression, migration, viability, and apoptosis were assessed using EdU/PI analysis, scratch assays, CCK‐8 assays, and Annexin V/PI flow cytometry.

**Results:**

GLI3 expression was enriched in THY1^+^CD34^−^ RASFs at both mRNA and protein levels. GLI3 KD increased GLI1 expression and upregulated genes involved in inflammation, matrix remodeling, and cell cycle regulation, including IL6, IL11, IL24, IL33, MMP3, PLAU, CCNA2, and E2F1. Pathway enrichment analysis revealed activation of ECM–receptor interaction, PI3K–Akt, and TNF signaling. Co‐silencing GLI1 with GLI3 blunted the induction of IL11, IL24, IL33, CCNA2, E2F1, and PLAU observed with GLI3 KD alone, indicating that GLI1 mediates a subset of the transcriptional effects induced by GLI3 loss. GANT61 suppressed CCNA2 and E2F1 expression, inhibited RASF proliferation and migration, and did not markedly increase apoptosis.

**Conclusion:**

GLI3 functions as a negative regulator of GLI1 and its downstream targets that drive the pathogenic behavior of RASFs. Targeting the GLI1–GLI3 axis may represent a promising therapeutic strategy to modulate fibroblast‐driven inflammation and joint destruction in RA.

**Abbreviations**
b/tsDMARDs: biologic or targeted synthetic disease‐modifying antirheumatic drugs
D2T RA: difficult‐to‐treat rheumatoid arthritis
DEGs: differentially expressed genes
GLI3‐FL: full‐length GLI3
GLI3‐R: repressor form of GLI3
HH: Hedgehog
KD: knockdown
qPCR: quantitative polymerase chain reaction
RA: rheumatoid arthritis
RASF: rheumatoid arthritis synovial fibroblast
RNA‐seq: RNA sequencing
siRNA: small interfering RNA
TNF‐α: tumor necrosis factor alpha

## 1 Introduction

Rheumatoid arthritis (RA) is a chronic autoimmune disorder characterized by persistent synovial inflammation, in which pathological interactions between immune and stromal cells—particularly synovial fibroblasts (SFs)—drive progressive joint destruction, including cartilage degradation and bone erosion [1, 2]. The advent of targeted therapies, such as inhibitors of pro‐inflammatory cytokines (e.g., TNF‐α and IL‐6) and small‐molecule inhibitors of the JAK–STAT signaling pathway, has markedly improved disease outcomes for many patients. However, a subset of individuals—estimated to be up to 20%—experience inadequate therapeutic responses or drug intolerance despite treatment with multiple classes of biologic or targeted synthetic disease‐modifying antirheumatic drugs (b/tsDMARDs), a condition now classified as difficult‐to‐treat RA (D2T RA) [3, 4, 5]. The molecular basis underlying this treatment resistance remains poorly understood, underscoring the urgent need for alternative therapeutic strategies.

Recent advances in transcriptomic profiling, including single‐cell RNA sequencing, have revealed substantial inter‐patient variability in the cellular composition of the inflamed synovium, which may significantly influence treatment responses. Notably, the R4RA trial—the first precision medicine trial in RA guided by synovial biopsy transcriptomics—unexpectedly highlighted the contribution of synovial fibroblast subsets to therapeutic resistance [6]. Specifically, synovial tissues enriched with fibroblast‐associated gene expression signatures were found to respond poorly to conventional immunosuppressive therapies, including IL‐6 receptor blockade and B cell–targeted therapies. These findings suggest that strategies directly targeting SF activity may complement current immunomodulatory approaches. Pathogenic rheumatoid arthritis synovial fibroblasts (RASFs) exhibit tumor‐like properties, such as unchecked proliferation, enhanced migratory and invasive potential, angiogenic support, and the secretion of inflammatory mediators that perpetuate chronic synovitis and joint destruction. Despite their pivotal role in RA pathogenesis, no approved therapies to date specifically target SFs, emphasizing the need to elucidate the molecular pathways governing their pathogenic behavior [7, 8].

To identify transcription factors (TFs) that drive the pathological features of SF subsets—including excessive proliferation, invasion, angiogenesis, and cytokine production—we previously performed microarray‐based transcriptomic profiling of the three major RASF subsets: THY1^−^CD34^−^ lining cells, THY1^+^CD34^−^ sublining cells, and THY1^+^CD34^+^ sublining cells [9, 10, 11, 12, 13]. These subsets are anatomically and functionally distinct, with THY1^−^CD34^−^ lining RASFs enriched for tissue‐destructive programs, THY1^+^CD34^−^ sublining RASFs showing prominent inflammatory and immune‐interacting features, and THY1^+^CD34^+^ sublining RASFs exhibiting a comparatively less inflammatory, stromal‐supportive phenotype [9, 10, 11, 12, 13]. More recent single‐cell and spatial studies suggest that these major subsets are further diversified by inflammation‐associated states, including HLA‐DRA^high^ RASFs with inflammatory and antigen‐presentation‐related features, ITGA5^+^ RASFs linked to proinflammatory niche formation, and COMP^hi^ fibrogenic RASFs associated with treatment resistance [14, 15, 16]. Against this evolving framework, GLI3 emerged as one of the most highly expressed transcription factors in the THY1^+^CD34^−^ sublining subset, which is consistently associated with aggressive fibroblast phenotypes.

**FIGURE 1:**
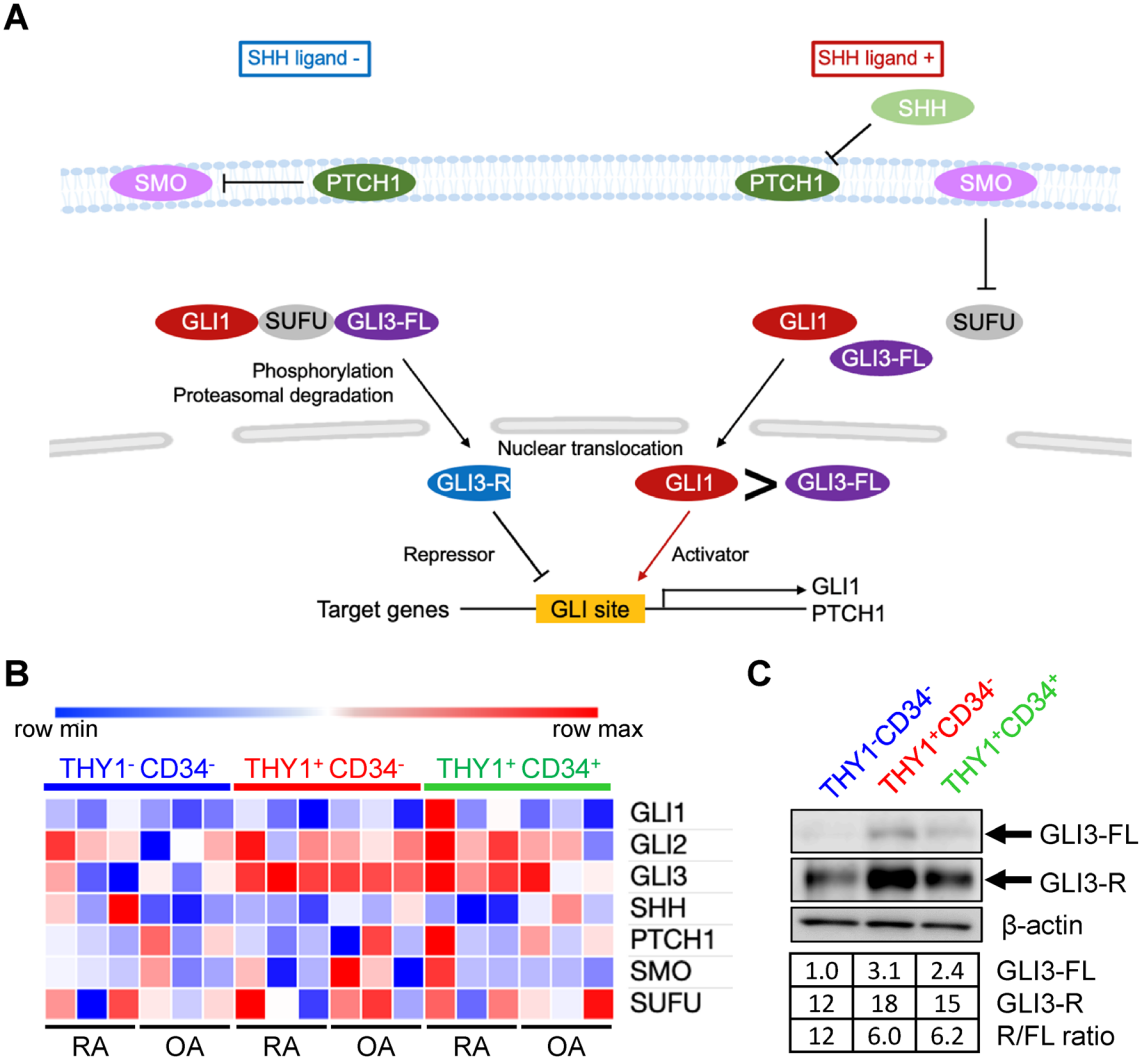
Expression of GLI3 protein in RASF subsets. (A) Schematic diagram of the Hedgehog–GLI signaling pathway. Canonical SHH–PTCH1 signaling promotes the nuclear translocation of the transcriptional activators GLI1, GLI2, and full‐length GLI3 (GLI3‐FL). In contrast, in the absence of ligand, GLI3‐FL undergoes proteolytic processing into the truncated repressor form GLI3‐R, thereby maintaining a balance between activation and repression within the HH–GLI pathway. (B) Heat map showing the expression of HH/GLI‐related components in three RASF subsets. Microarray data from THY1^−^CD34^−^, THY1^+^CD34^−^, and THY1^+^CD34^+^ subsets derived from three RA and three OA patients were previously published [9, 13]. Among the components, only GLI3 was significantly upregulated in the THY1^+^CD34^−^ subset (log_2_FC > 0.5, *p* < 0.05, FDR < 0.01). (C) Western blotting analysis of GLI3 in freshly isolated RASF subsets. Full‐length GLI3 (GLI3‐FL, 190 kDa) and repressor GLI3 (GLI3‐R, 83 kDa) are indicated by arrows. β‐Actin was used as a loading control. Band intensities were quantified using ImageJ, normalized to β‐Actin, and expressed as fold changes relative to the THY1^−^CD34^−^ subset, whose GLI3‐FL level was set to 1.0.

The Hedgehog (HH)–GLI signaling pathway is an evolutionarily conserved regulatory cascade that orchestrates embryonic development, tissue patterning, and cellular proliferation, differentiation, and oncogenesis [17]. In mammals, the three GLI transcription factors—GLI1, GLI2, and GLI3—mediate the transcriptional outputs of HH signaling. GLI1 primarily acts as a transcriptional activator, whereas GLI2 and GLI3 may function as either full‐length activators (FL) or as truncated repressors (R), depending on their post‐translational processing. In the absence of HH ligands, GLI3 is phosphorylated and partially degraded to yield GLI3‐R, which acts as a repressor by competitively binding to GLI target sequences and antagonizing GLI1 activity. Upon HH ligand stimulation, this repression is relieved through the degradation of GLI3‐R and/or the stabilization of GLI3‐FL, thereby enabling transcriptional activation of HH target genes [17, 18] (Figure 1A).

While previous studies have demonstrated upregulated expression of HH pathway components—particularly GLI1—in RA synovial tissue [19, 20, 21, 22, 23, 24], the role of GLI3 in RASFs remains largely unexplored. Intriguingly, recent studies in cancer have revealed that GLI3 can regulate cellular proliferation and invasion through non‐canonical, HH‐independent pathways, suggesting that its effects are context‐dependent [25]. In the present study, we aimed to elucidate the functional role of GLI3, the most highly expressed TF in the aggressive THY1^+^CD34^−^ sublining RASF subsets. Our findings uncover a reciprocal GLI3–GLI1 regulatory axis and suggest that targeting this pathway may represent a promising strategy to suppress fibroblast‐driven inflammation and joint damage in RA.

## 2 Materials and Methods

### 2.1 Patient Recruitment

This study was conducted in accordance with the Declaration of Helsinki and approved by the Medical Research Ethics Committee of the Institute of Science Tokyo (M2000‐979); synovial tissue was obtained from RA patients undergoing arthroplasty after written informed consent [13].

### 2.2 Synovial Cell Isolation and Culture

After mincing, synovial tissues were enzymatically digested in DMEM at 37°C using 2 mg/mL collagenase type IV (Worthington, NJ, USA), 0.8 mg/mL dispase II, and 0.1 mg/mL DNase I (Roche, Basel, Switzerland) with gentle agitation. The supernatant was collected every 15 min and replaced with fresh enzyme solution four times. After ACK lysis, cells were processed along two predefined routes:

Subset‐sorted SFs (ex vivo) for Western blotting and microarray: Live CD45^−^CD235a^−^CD31^−^PDPN^+^ cells were sorted into THY1^−^CD34^−^, THY1^+^CD34^−^, and THY1^+^CD34^+^ subsets using FACSAria III (BD Biosciences, CA, USA) as previously described [9, 12, 13], and the gating strategy of SF subsets was shown in Figure S1. These cells were used without in vitro passaging for Western blotting. Microarray data from our previous work [9, 13] were reanalyzed for the expression of HH‐GLI pathway component genes.

Bulk RASFs (cultured) for RNA‐seq and functional assays: Cell suspensions were plated in DMEM supplemented with 10% FBS and penicillin/streptomycin and passaged weekly to avoid overconfluence. Cells at passages 6–10 were used for RNA‐seq and functional assays, including scratch migration, EdU incorporation, and cell cycle analysis, Annexin V/PI apoptosis assays, and cell viability assays.

### 2.3 Antibodies and Reagents

The following reagents and antibodies were used for the analysis of synovial cells with flow cytometry, cell sorting, and several in vitro assays: anti‐CD45‐APC‐H7 (2D1, BD Biosciences, CA, USA), anti‐CD235a‐APC‐Alexa Fluor750 (11E4B‐7‐6, Beckman Coulter, FL, USA), anti‐CD31‐PE‐Cyanine7 (WM‐59, eBioscience, CA, USA), anti‐CD146‐APC (P1H12, eBioscience), anti‐CD34‐PE (4H11, eBioscience), anti‐PDPN‐PerCP‐eFluor710 (NZ‐1.3, eBioscience), anti‐THY1‐FITC (5E10, BD Biosciences), anti‐CD73‐PE‐CF594 (AD2, BD Biosciences), anti‐CD271‐APC (ME20.4, eBioscience), anti‐CD54‐PE‐CF594 (HA58, BioLegend, CA, USA), anti‐CD44‐APC (G44‐26, BD Biosciences), anti‐CD29‐APC (TS2/16, BioLegend), human TruStain FcX (BioLegend), Live/Dead fixable aqua dead cell stain kits (Molecular Probes, Thermo Fisher Scientific, MA, USA), anti‐GLI3 (AF3690, R&D Systems, Minneapolis, MN, USA), anti‐β‐actin (AC‐15, Sigma‐Aldrich, St. Louis, MO, USA), horseradish peroxidase (HRP)‐conjugated mouse IgG and HRP‐conjugated rabbit IgG (7074 and 7076, Cell Signaling Technology, Danvers, MA, USA), and the GLI1 inhibitor GANT61 (HY‐13901, MedChemExpress, Monmouth Junction, NJ, USA).

### 2.4 Gene Knockdown by siRNA

Bulk RASFs were seeded onto plates and transfected with siRNA using Lipofectamine RNAiMAX (Thermo Fisher Scientific, Waltham, MA, USA) according to the manufacturer's instructions. After 72 h, the transfected cells were stimulated with 10 ng/mL TNF‐α for 24 h. The siRNAs used were siGLI1 (Thermo Fisher Scientific, Silencer Select: s5815), siGLI3 (Thermo Fisher Scientific, Silencer Select: s5822), and siCtrl (Thermo Fisher Scientific, Silencer Select Negative Control siRNA No. 2). The siRNA knockdown efficiencies are shown in Figure S2.

### 2.5 RNA‐Sequencing Analysis

Poly(A)^+^ RNA was extracted from siGLI3‐, siGLI1‐ and siCtrl‐transfected bulk RASF derived from three independent RA donors (*n* = 3 biological replicates per group) using the RNeasy Mini Kit (Qiagen, Hilden, Germany). Strand‐specific mRNA libraries were prepared with the BGI Optimal Dual‐mode mRNA Library Prep Kit according to the manufacturer's protocol. In brief, poly(A)^+^ RNA was captured with oligo(dT) beads, fragmented, converted to cDNA, and second‐strand synthesis incorporated dUTP to preserve strandedness. After end repair, A‐tailing, adaptor ligation, PCR amplification, and quality control with an Agilent 2100 Bioanalyzer, double‐stranded libraries were circularized, amplified by Φ29 rolling‐circle replication to generate DNA nanoballs (~300 copies each), and sequenced as paired‐end 100‐bp reads on a DNBSEQ‐G400 platform (BGI, Hong Kong). Raw reads were filtered with SOAPnuke v2.2.1 to remove adaptors and low‐quality or ambiguous bases, and clean reads were aligned to the GRCh38.p12 human reference genome using Bowtie2 v2.4.5, and gene‐level read counts and expression values (TPM, FPKM) were obtained with RSEM v1.3.1. For differential expression analysis, the gene‐by‐sample read‐count matrix from RSEM was imported into DESeq2 v1.34.0, and genes with a false discovery rate (FDR) ≤ 0.05 were considered significantly differentially expressed. TPM and FPKM values were used only for descriptive purposes (e.g., visualization). The full read‐count matrix used as DESeq2 input is provided in Table S1. Volcano plots were generated using GraphPad Prism 10. Functional enrichment of differentially expressed genes (DEGs) for Gene Ontology (GO) and KEGG categories was assessed using the hypergeometric test (R function phyper), and Benjamini–Hochberg–adjusted *Q*‐values ≤ 0.05 were considered significant. RNA‐seq data is provided in Table S1.

### 2.6 Real‐Time Quantitative Polymerase Chain Reaction

RNA was extracted from bulk RASFs using the RNeasy Mini Kit (Qiagen, Hilden, Germany), and complementary DNA (cDNA) was synthesized using the QuantiTect Reverse Transcription Kit (Qiagen). Quantitative polymerase chain reaction (qPCR) was performed on a LightCycler 96 (Roche) using SYBR Green PCR Master Mix (Qiagen). Primer sequences for all genes analyzed are listed in Table S2.

### 2.7 Western Blotting

Freshly isolated RASF subsets were lysed in 2% SDS sample buffer containing complete mini protease inhibitor (Roche) and phosphatase inhibitor cocktail (Sigma‐Aldrich). Cell lysates were boiled with 5% 2‐mercaptoethanol at 95°C for 5 min and subjected to SDS‐PAGE. The PVDF membrane was blocked with 5% bovine serum albumin in TBS‐T for 1 h at room temperature, incubated overnight at 4°C with primary antibodies (GLI3: 1:1000, β‐actin: 1:5000) in Can Get Signal Solution 1 (NKB‐301, TOYOBO, Osaka, Japan), and then incubated with HRP‐conjugated secondary antibody (1:5000) in Can Get Signal Solution 2 (NKB‐301, TOYOBO). Signals were detected using ECL Prime Western blotting Detection Reagent (Amersham Biosciences, GE Healthcare, Little Chalfont, UK) and ImageQuant LAS‐4000 (FUJIFILM Life Science, Tokyo, Japan). Band intensities of GLI3‐FL and GLI3‐R were quantified using ImageJ (NIH, Bethesda, MD, USA) following a standard protocol. Mean gray values were background‐corrected, normalized to β‐actin in the same lane, and expressed as fold changes relative to the THY1^−^CD34^−^ subset, whose GLI3‐FL level was set to 1.0.

### 2.8 Scratch Assay

To assess cell migration ability, bulk RASFs (1.0 × 10^5^ cells/mL) were plated in 1 mL DMEM in a 12‐well plate. After overnight incubation in 5% CO_2_ at 37°C, a sterile 1000 μL pipette tip was used to create a scratch in the cell monolayer. The cells were washed with PBS to remove debris, and fresh DMEM was added. Two days before harvest, the cells were pretreated with 0, 5, or 10 μM GANT61, followed by stimulation with 0 or 10 ng/mL TNF‐α for 24 h. Images were captured using BZ‐9000 All‐in‐One Fluorescence Microscope (KEYENCE, Osaka, Japan), and the closure rate was analyzed using ImageJ (NIH, Bethesda, MD, USA).

### 2.9 EdU Incorporation and Cell Cycle Analysis

To evaluate DNA synthesis and cell cycle distribution, bulk RASFs were cultured under the same conditions and treatment schedule as described for the scratch assay. 20 μM EdU was added together with TNF‐α for the final 24 h. Cells were then harvested and stained using the Click‐iT Plus EdU Alexa Fluor 647 Flow Cytometry Assay Kit (C10634, Invitrogen, Carlsbad, CA, USA) and FxCycle PI/RNase Staining Solution (F10797, Invitrogen, Carlsbad, CA, USA). Flow cytometric data were acquired using FACSLyric (BD Biosciences, CA, USA) and analyzed to determine EdU incorporation and cell cycle distribution (G0/G1, S, and G2/M phases) based on EdU incorporation and DNA content. Relative EdU incorporation was calculated by normalizing to the untreated control and used for figure presentation.

### 2.10 Annexin V/PI Apoptosis Assay

To assess apoptosis, bulk RASFs were cultured under the same conditions and treatment schedule as described for the scratch assay, and apoptosis was evaluated in TNF‐α‐stimulated cells with or without GANT61. Untreated cells served as a negative control, and cells treated with 10 μg/mL actinomycin D plus 10 ng/mL TNF‐α for 24 h served as a positive control. Cells were then harvested and stained using the Dead Cell Apoptosis Kit with Annexin V Alexa Fluor 488 and Propidium Iodide (PI) (Thermo Fisher Scientific, Waltham, MA, USA). Flow cytometric data were acquired using FACSLyric and analyzed to determine the proportions of viable, apoptotic, and necrotic cells.

### 2.11 Cell Viability Assay

To evaluate cell viability, bulk RASFs were cultured under the same treatment schedule as described for the scratch assay, except that cells were seeded in 96‐well plates. Cell viability was assessed using the Cell Counting Kit‐8 (Dojindo Molecular Technologies, Rockville, MD, USA). Relative viability was calculated by normalizing to the untreated control and used for figure presentation.

### 2.12 Statistical Analysis

Data in bar graphs are presented as mean ± standard deviation (SD). For comparisons between two matched groups, paired *t*‐tests were used. For comparisons among three or more matched groups, one‐way ANOVA followed by Bonferroni's multiple‐comparison test was performed. A *p* < 0.05 was considered statistically significant. Statistical analyses were performed using Prism 10 (GraphPad Software).

## 3 Results

### 3.1 GLI3 Is Preferentially Expressed in the THY1 ^+^ CD34 ^−^ RASF Subset

We previously compared the mRNA expression levels of TFs across three major SF subsets obtained from 3 RA and 3 osteoarthritis (OA) patients—THY1^−^CD34^−^, THY1^+^CD34^−^, and THY1^+^CD34^+^—to identify regulators involved in IL‐6 production [13]. Among 65 differentially expressed TFs, GLI3 was significantly upregulated in the THY1^+^CD34^−^ sublining subset, which is associated with aggressive pathogenic behavior (Figure 1B). GLI3 is a transcription factor functioning downstream of the Hedgehog (HH)–GLI signaling pathway and can act either as a full‐length activator (GLI3‐FL) or as a truncated repressor (GLI3‐R), depending on the presence of HH ligands (Figure 1A) [17]. Consistent with these findings, re‐analysis of a publicly available single‐cell RNA‐seq dataset of RA synovial tissue [11] showed that GLI3 expression was preferentially enriched in the THY1^+^ sublining RASF subsets from patients with clinically active RA, whereas those in sustained remission exhibited lower GLI3 expression (Figure S3).

Interestingly, in our microarray dataset, other HH–GLI pathway components—including SHH, SMO, SUFU, PTCH1, and other GLI family members (GLI1, GLI2)—showed no statistically significant differences in expression among the SF subsets (Figure 1B). This suggests that the upregulation of GLI3 in the THY1^+^CD34^−^ subset may occur largely independently of canonical HH pathway activation.

We further evaluated GLI3 protein expression in freshly isolated RASF subsets from RA synovial tissue (Figure 1C and Figure S6). Because GLI3‐R (83 kDa) is generated by post‐translational, PKA/GSK3β‐dependent proteolysis of GLI3‐FL (190 kDa), its abundance cannot be inferred from transcriptomic analysis. We therefore assessed the expression of GLI3‐FL and GLI3‐R by Western blotting with an antibody recognizing the common N‐terminal domain of both forms. Both GLI3‐FL and GLI3‐R were more abundantly expressed in the THY1^+^CD34^−^ subset compared to the THY1^−^CD34^−^ and THY1^+^CD34^+^ subsets. Notably, GLI3‐R was the predominant detectable form in all subsets, while both GLI3‐FL and GLI3‐R were most abundant in the THY1^+^CD34^−^ subset. These results indicate that both GLI3‐FL and GLI3‐R were highly expressed in the pathogenic THY1^+^CD34^−^ subset.

### 3.2 GLI3 Represses Pro‐Inflammatory and Proliferative Gene Programs in RASFs

**FIGURE 2:**
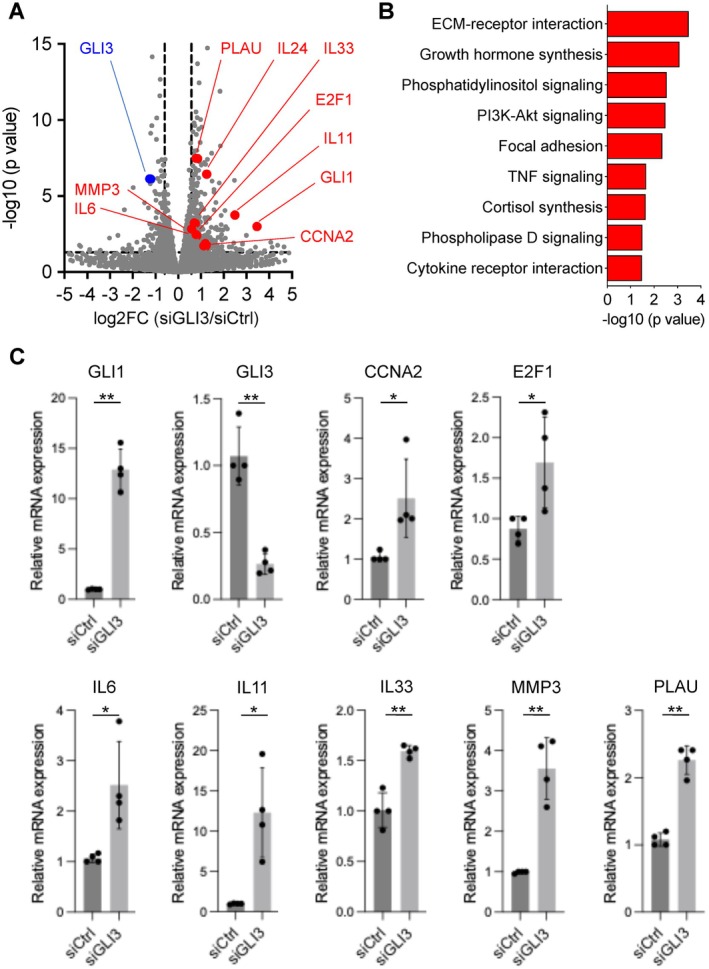
Identification of GLI3 target genes by RNA‐seq in RASFs. (A) Volcano plot showing differentially expressed genes between siCtrl‐ and siGLI3‐transfected RASFs (log_2_FC > 0.5, FDR < 0.05). Bulk RASFs derived from three independent RA donors were transfected with siCtrl or siGLI3, followed by stimulation with 10 ng/mL TNF‐α for 24 h after 72 h of transfection. Total RNA was subjected to bulk RNA‐seq, and differential expression was assessed using DESeq2 v1.34.0 on the RSEM‐derived read‐count matrix (*n* = 3). (B) KEGG pathway enrichment analysis of genes upregulated by siGLI3 (DESeq2, siGLI3 vs. siCtrl). The top nine significantly enriched pathways (Benjamini–Hochberg–adjusted *Q* < 0.05) are shown. (C) qPCR validation of selected differentially expressed genes identified by RNA‐seq in an independent set of bulk RASF samples (*n* = 4 biological replicates). Gene expression levels were normalized to the internal control and are presented relative to the siCtrl group. Data represent mean ± SD. *p < 0.05, **p < 0.01 by paired *t*‐test.

To investigate the functional role of GLI3 in RASFs treated with TNF‐α, we performed siRNA‐mediated knockdown (KD) of GLI3 followed by RNA‐seq analysis. Among the genes upregulated upon GLI3 KD, GLI1, a canonical target and effector of the HH–GLI pathway, exhibited one of the largest fold changes (Figure 2A). In addition, genes associated with the pathogenic behavior of RASFs—including IL6, IL11, IL24, IL33, CCNA2, E2F1, MMP3, and PLAU—were also significantly upregulated in GLI3‐deficient cells (Figure 2A).

KEGG pathway enrichment analysis of the upregulated gene set revealed significant enrichment in pathways involved in ECM–receptor interaction, PI3K–Akt signaling, focal adhesion, TNF signaling, and cytokine–cytokine receptor interaction (Figure 2B).

qPCR analysis in an independent set of samples confirmed the same directional changes in selected DEGs identified by RNA‐seq (Figure 2C). These results suggest that GLI3 functions primarily as a transcriptional repressor in RASFs, suppressing genes associated with inflammatory, proliferative, and migratory behavior.

### 3.3 GLI1 Mediates a Subset of Gene Induction Following GLI3 Knockdown

**FIGURE 3:**
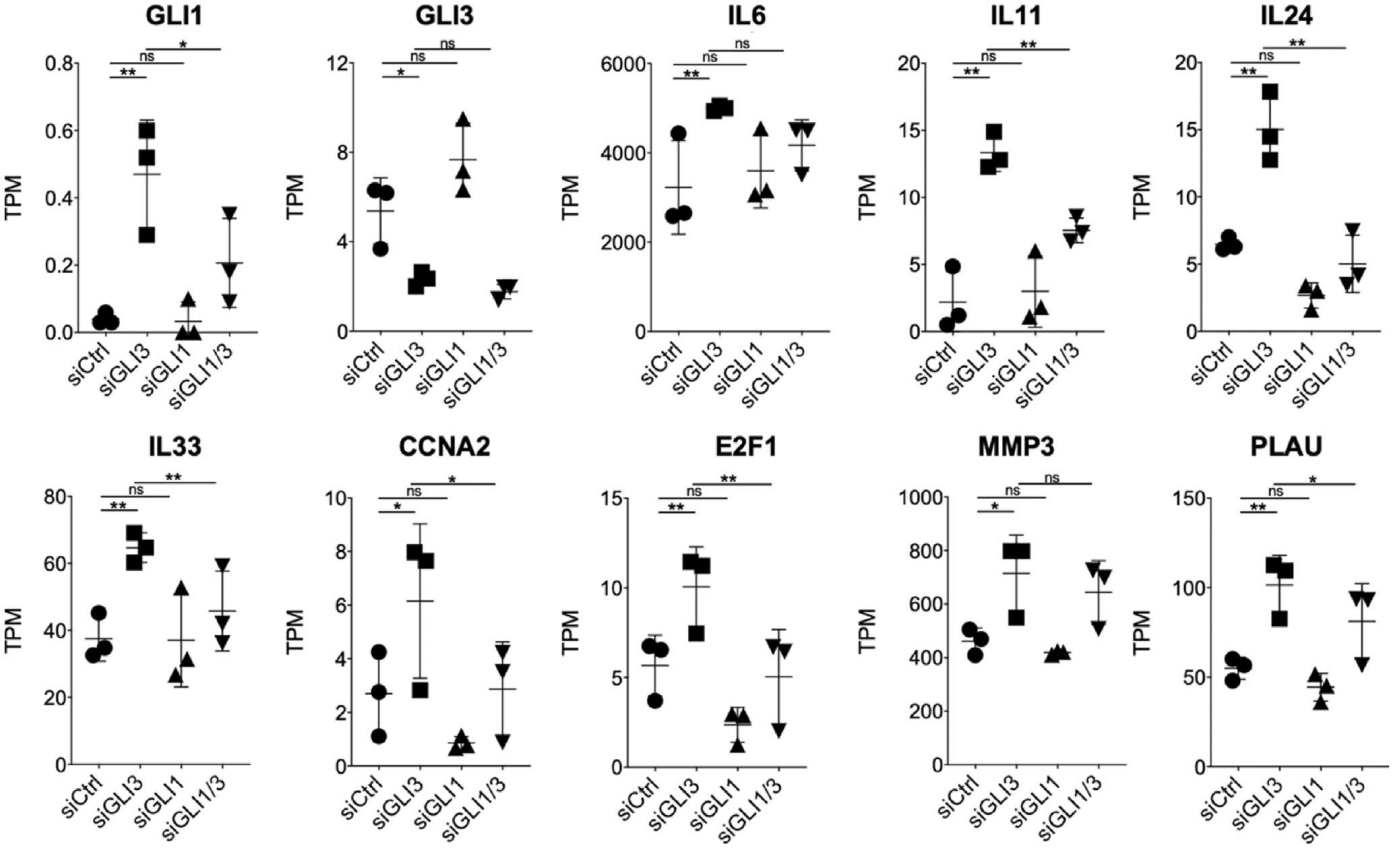
Involvement of GLI1 in GLI3‐mediated gene regulation in RASFs. Bulk RASFs derived from three independent RA donors were transfected with siCtrl, siGLI3, siGLI1, or a combination of siGLI3 and siGLI1, followed by stimulation with 10 ng/mL TNF‐α for 24 h after 72 h of transfection. Total RNA was analyzed by bulk RNA‐seq, and gene expression was quantified as TPM using RSEM v1.3.1. Bar graphs summarize RSEM‐derived TPM values (mean ± SD; *n* = 3) for selected GLI3‐mediated genes identified in the RNA‐seq analysis. Statistical significance was determined by one‐way ANOVA with Bonferroni's multiple comparison test. *p < 0.05, **p < 0.01; ns, not significant.

Given the strong upregulation of GLI1 and multiple pathogenic genes upon GLI3 KD, we hypothesized that GLI1 may mediate the transcriptional effects induced by GLI3 loss. To test this, we performed RNA‐seq following siRNA knockdown of GLI1 alone, and dual knockdown of GLI3 and GLI1. Knockdown of GLI1 alone had minimal impact on gene expression, likely due to its low basal expression in RASFs (Figure 3). In contrast, the co‐silencing of GLI1 with GLI3 significantly attenuated the induction of IL11, IL24, IL33, CCNA2, E2F1, and PLAU observed with GLI3 KD alone (Figure 3), suggesting that these genes are positively regulated by GLI1. In contrast, the expression of IL6 and MMP3, while elevated upon GLI3 KD, remained unaffected by the additional knockdown of GLI1, indicating a GLI1‐independent regulatory mechanism.

These findings indicate that GLI1 acts as a transcriptional activator for a subset of GLI3‐repressed genes, and that the GLI3–GLI1 axis plays a selective role in the regulation of inflammatory and proliferative genes in RASFs.

### 3.4 Pharmacological Inhibition of GLI1 Suppresses RASF Proliferation and Migration

**FIGURE 4:**
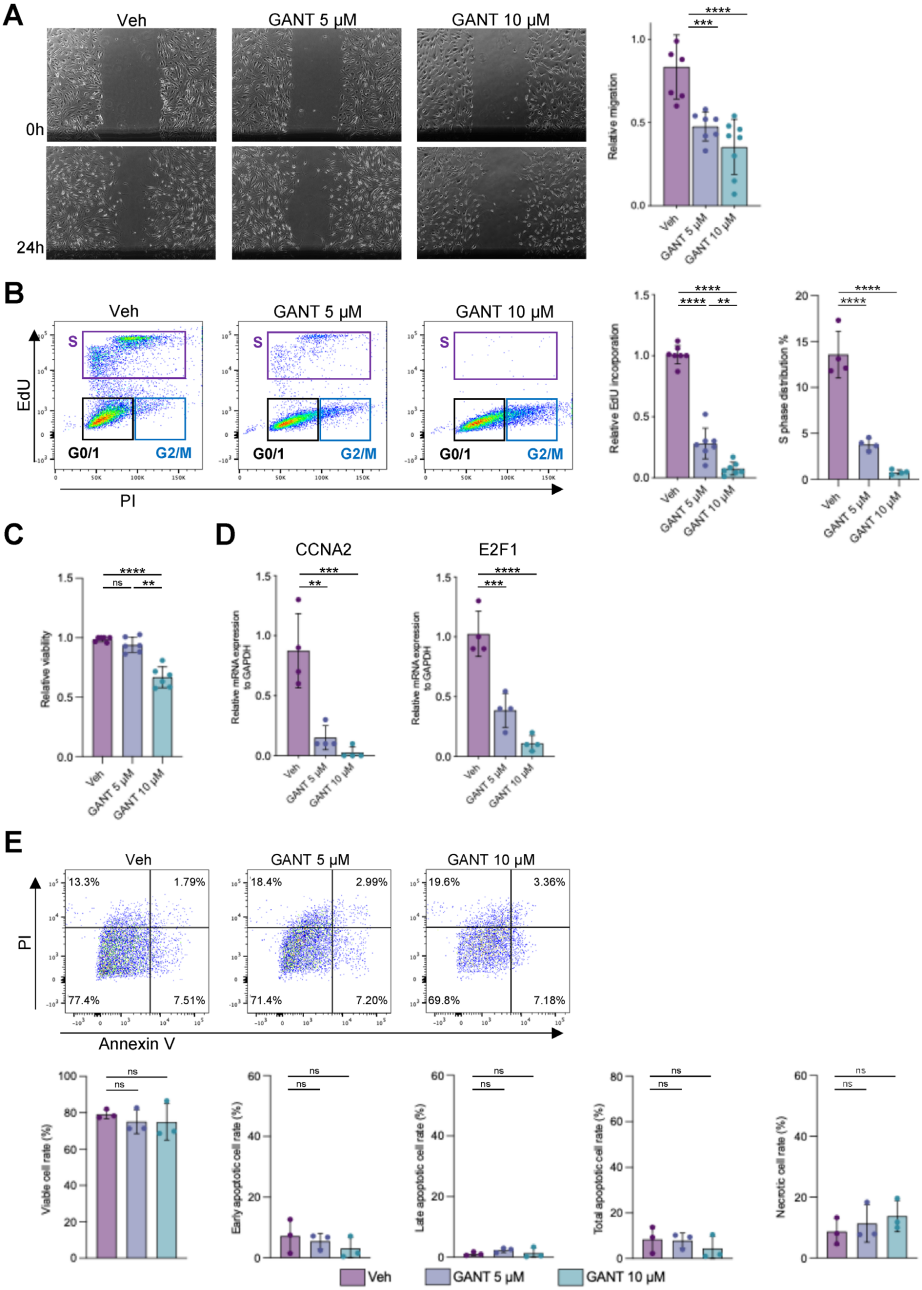
Suppression of RASF proliferation and migration by the GLI1 inhibitor GANT61. (A) Scratch assay of bulk RASFs treated with GANT61. Representative images and quantification of relative migration are shown (*n* = 4 biological replicates). (B) EdU incorporation and cell cycle analysis of bulk RASFs treated with GANT61. Representative plots and quantification of relative EdU incorporation and S‐phase fraction are shown (*n* = 4 biological replicates). (C) Cell viability of bulk RASFs treated with GANT61, as assessed by CCK‐8 assay (*n* = 3 biological replicates). (D) qPCR analysis of CCNA2 and E2F1 expression in bulk RASFs treated with GANT61 (*n* = 4 biological replicates). (E) Annexin V/PI apoptosis assay of bulk RASFs treated with GANT61. Representative plots and quantification of viable, early apoptotic, late apoptotic, total apoptotic, and necrotic cell fractions are shown (*n* = 3 biological replicates). Data are presented as mean ± SD. Statistical significance was assessed by one‐way ANOVA followed by Bonferroni's multiple comparison test. *p < 0.05, **p < 0.01, ***p < 0.001, ****p < 0.0001.

The observation that GLI1 KD showed a modest suppressive effect on CCNA2, E2F1, and PLAU—genes involved in cell proliferation and migration—suggested a role for GLI1 in promoting the aggressive phenotype of RASFs. To evaluate whether pharmacological inhibition of GLI activity could reproduce these effects, we treated RASFs with GANT61, a small‐molecule inhibitor of GLI1 transcriptional activity [26]. Scratch assays showed that GANT61 significantly impaired RASF migration (Figure 4A). Consistently, EdU/PI cell‐cycle analysis demonstrated a dose‐dependent reduction in EdU incorporation and in the S‐phase fraction, indicating suppressed proliferative activity (Figure 4B and Figure S4). At 5 μM, GANT61 did not significantly reduce cell viability in CCK‐8 assays (Figure 4C). In parallel, GANT61 decreased the expression of the cell‐cycle–associated genes CCNA2 and E2F1 (Figure 4D). Moreover, Annexin V/PI analysis showed no significant increase in total apoptosis under these conditions (Figure 4E and Figure S5).

Taken together, these results suggest that GLI1—normally repressed by GLI3 in RASFs—contributes to the proliferative and migratory phenotype of RASFs, and that pharmacological GLI inhibition suppresses these pathogenic properties without marked apoptosis induction.

## 4 Discussion

**FIGURE 5:**
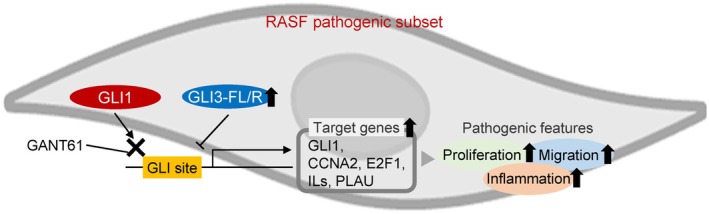
Hypothetical model of the reciprocal regulation between GLI1 and GLI3 in pathogenic RASFs. In the pathogenic RASF subset, GLI3‐FL/R is highly expressed and inhibits GLI1 activity via competitive binding to GLI sites, thereby suppressing the transcription of GLI1 target genes involved in proliferation, migration, and inflammation. GANT61 inhibits GLI1‐DNA binding and may serve as a therapeutic agent targeting RASF‐mediated pathology.

In this study, we identified a reciprocal regulatory relationship between GLI1 and GLI3 in the aggressive behaviors of RASFs. While previous studies have demonstrated that GLI1 promotes the inflammatory and invasive characteristics of RASFs, and that its inhibition ameliorates RA pathology in both in vitro and in vivo models [27, 28, 29], our findings identify GLI3—which is highly expressed in the pathogenic subset—as a negative regulator of GLI1 activity in RASFs. These results expand our understanding of GLI family transcriptional dynamics and suggest that dysregulated GLI1–GLI3 interactions may contribute to RASF pathogenicity, presenting a potential therapeutic target (Figure 5).

Among the transcription factors identified by microarray‐based profiling across SF subsets, GLI3 was one of the few significantly upregulated in the THY1^+^CD34^−^ population, a subset particularly associated with inflammatory pathogenic features in RA. This interpretation should be integrated with the current view that the pathogenic sublining RASF subsets contain multiple functionally distinct states, including inflammatory, immune‐interacting, and fibrogenic populations [14, 15, 16]. While GLI1 expression is well known to be induced by canonical HH/GLI signaling, the regulatory mechanisms governing GLI3 expression are far less understood. Recent studies have reported elevated GLI3 expression in various cancer cell types, driven by epigenetic mechanisms such as promoter methylation and post‐transcriptional regulation by specific microRNAs [25]. Additionally, developmental studies have implicated Wnt/β‐catenin/Tcf and Notch/RBPJ signaling pathways in the transcriptional activation of GLI3 during neural differentiation [30, 31, 32]. Given that both Wnt and Notch signaling are involved in the differentiation and activation of RASF subsets, it is plausible that these pathways contribute to the elevated GLI3 expression observed in the pathogenic THY1^+^CD34^−^ subset. Moreover, recent work in innate immune cells has shown that GLI3 can be induced by LPS via the TLR4–TRIF–IRF3 axis [33]. It remains possible that pro‐inflammatory cytokines and growth factors involved in RASF activation contribute to GLI3 upregulation. These observations raise the possibility that elevated GLI3 expression in the pathogenic subset reflects a negative feedback mechanism aimed at restraining excessive GLI1‐driven transcriptional activity. It is also possible that the GLI1–GLI3 axis is not uniform within the broader THY1^+^CD34^−^ RASF subset, which may encompass functionally distinct fibroblast states. Further subdivision of this subset may therefore help clarify whether GLI3‐mediated regulation differs among pathogenic RASF subpopulations.

GLI3 can function either as a transcriptional activator (GLI3‐FL) or repressor (GLI3‐R), depending on the presence or absence of HH ligands. In the context of canonical HH signaling, its primary role is to repress HH target genes under basal conditions, and GLI3 is preferentially processed into the repressor form under ligand‐poor conditions. Although this study did not directly determine the mechanism underlying the predominance of GLI3‐R over GLI3‐FL in the THY1^+^CD34^−^ subset, our results are compatible with a state in which canonical HH ligand input is limited. However, recent evidence from cancer research suggests that GLI3 may also exhibit oncogenic functions, promoting tumor cell proliferation and invasion [25]. For instance, in prostate cancer cells, GLI3‐R has been shown to promote proliferation through interaction with the androgen receptor [34]. According to our siRNA experiments in RASFs, GLI3 acted predominantly as a transcriptional repressor of GLI1 target genes, consistent with its canonical function. This suggests that the THY1^+^CD34^−^ subset may retain responsiveness to HH ligands. In addition to canonical HH signaling, several other pathways—including PKA, GSK3β, and Notch—have been shown to regulate GLI3 processing and shift the balance between its repressor and activator forms [25]. These non‐canonical inputs may also contribute to fibroblast activation by modulating the GLI1–GLI3 axis. Although GLI2 expression did not differ significantly among SF subsets in our data, previous studies have shown that GLI2 mediates fibroblast activation and tissue fibrosis, particularly in response to TGF‐β signaling, suggesting that GLI2 may modulate RASF behavior under specific inflammatory or fibrotic conditions.

Our data using GANT61, a selective GLI1 inhibitor, further support the critical role of GLI1 in regulating RASF proliferation and migration. In our in vitro system, GANT61 suppressed migration, EdU incorporation, S‐phase entry, and the expression of CCNA2 and E2F1, without marked apoptosis induction. The lack of a significant viability decrease at 5 μM further suggests that these inhibitory effects were not merely due to nonspecific toxicity. GANT61 has been shown to reduce disease severity in collagen‐induced arthritis (CIA) models by inhibiting cell proliferation and inducing apoptosis [35], partly consistent with our in vitro findings. Similarly, cyclopamine, a classical HH pathway inhibitor, has been reported to suppress SHH, PTCH1, SMO, and GLI1 expression, induce G1 cell cycle arrest, and inhibit RhoA/Rac1 signaling and cyclin D1 expression in RASFs [36, 37]. These effects contribute to impaired RASF migration and enhanced cartilage protection in antigen‐induced arthritis (AIA) models [19]. Currently, a range of HH/GLI pathway inhibitors—including HH ligand blockers, PTCH and SMO antagonists, and GLI transcriptional inhibitors—are under investigation in oncology [38]. Our findings support the translational relevance of the GLI1–GLI3 regulatory axis in RA and provide a rationale for exploring GLI1‐specific inhibition as a novel therapeutic approach targeting fibroblast‐driven pathology.

This study has several limitations. First, the precise molecular mechanisms underlying GLI3 upregulation in specific RASF subsets remain incompletely defined. Second, due to inter‐patient heterogeneity in RA, the relatively small sample size may not fully capture the diversity of disease subtypes. Third, functional assays were performed using cultured bulk RASFs, which may obscure subset‐specific effects and limit the extrapolation to in vivo contexts. Lastly, although our findings are consistent with non‐canonical regulation of GLI3, we did not directly examine the effects of canonical Hedgehog (HH) ligand stimulation and therefore cannot fully exclude its potential involvement. It is also possible that GLI1 is upregulated in the pathogenic subset via HH signaling in certain RA cases. Future studies incorporating single‐cell analyses and ligand‐based perturbations will be necessary to further clarify the regulation and function of GLI3 in RA pathogenesis.

## 5 Conclusion

We identified GLI3 as a negative regulator of GLI1 in RASFs. Dysregulated activity of GLI family transcription factors contributes to the overproduction of inflammatory mediators, as well as to the aberrant proliferation and migration of RASFs. These findings highlight the GLI1–GLI3 reciprocal regulatory axis as a novel pathogenic mechanism and a promising therapeutic target in RA.

## Author Contributions

Motohiko Sato, Yoji Komiya, Fumitaka Mizoguchi, Tetsuya Saito, and Shinsuke Yasuda conceived the study aims and design. Motohiko Sato, Yoji Komiya, and Tetsuya Saito performed experiments. Motohiko Sato, Yoji Komiya, Seiji Noda, Yasuhiro Tagawa, Akio Yamamoto, and Hideyuki Iwai contributed to synovial specimen processing as part of the core research team. Kentaro Endo, Hideyuki Koga, Yasuhiro Takahara, Kazutaka Sugimoto, and Ichiro Sekiya served as site investigators responsible for clinical synovial specimen provision. Eiryo Kawakami conducted statistical analyses. Motohiko Sato, Tadashi Hosoya, Tetsuya Saito, and Shinsuke Yasuda analyzed and interpreted the data. Motohiko Sato, Tetsuya Saito, and Shinsuke Yasuda drafted the manuscript; all authors contributed to discussion, critically reviewed the manuscript for important intellectual content, and approved the final version. (All authors meet ICMJE authorship criteria).

## Funding

This work was supported by the Japan Agency for Medical Research and Development (AMED) under grant number 21ek0410061h0003 and by the Japan Society for the Promotion of Science (JSPS) KAKENHI under grant numbers 21K20755 and 22K08519.

## Disclosure

T.S. received research funding from Eli Lilly, speaking fees from Abbvie, Asahi Kasei Pharma, Chugai Pharmaceutical, Eisai, Eli Lilly, GlaxoSmithKline, Mitsubishi Tanabe Pharma, Ono Pharmaceutical, Taisho Pharmaceutical, and Astellas Pharma. S.Y. received research funding from Asahi Kasei Pharma, Ayumi Pharmaceutical Corporation, Chugai Pharmaceutical, Nippon Boehringer Ingelheim Co. Ltd., and Taisho Pharmaceutical Co. Ltd., speaking fees from Abbvie, Asahi Kasei Pharma, Chugai Pharmaceutical, Eisai, Eli Lilly, GlaxoSmithKline, Mitsubishi Tanabe Pharma, and Ono Pharmaceutical, and consultant fees from Eisai, ImmunoForge, Novartis, and Otsuka Pharmaceutical Co. Ltd.

## Ethics Statement

Ethics approval and consent to participate. We have confirmed the ethics approval from the medical research ethics committee of Institute of Science Tokyo (approval number: M2000‐979).

## Consent

We have obtained written informed consent for publication from all the patients.

## Conflicts of Interest

The authors declare no conflicts of interest.

## Supporting information


**Figure S1:** The gating strategy of RASF subsets.
**Figure S2:** siRNA knockdown efficiencies.
**Figure S3:** scRNA‐seq Heatmap.
**Figure S4:** Full cell‐cycle distribution of bulk RASFs treated with GANT61 and TNF‐α.
**Figure S5:** Negative and positive control results for Annexin V/PI apoptosis analysis in bulk RASFs.
**Figure S6:** Uncropped Western blot images for GLI3 detection in freshly isolated RASF subsets.

**Table S1:** Gene‐by‐sample RNA‐seq read‐count matrix of bulk RASFs used for differential expression analyses in Figures 2 and 3.

**Table S2:** qPCR primer sequences.

## Data Availability

The microarray dataset analyzed in this study is publicly available in the Gene Expression Omnibus under accession number GSE109450. The bulk RNA‐seq dataset generated in this study has been deposited in the Gene Expression Omnibus under accession number GSE329806. The gene‐by‐sample bulk RNA‐seq read‐count matrix generated in this study is provided in Table S1. Additional data supporting the findings of this study are available from the corresponding author upon reasonable request.
