# Supplementary material for: Reciprocal Regulation of GLI1 and GLI3 Fine Tunes the Pathogenic Behavior of Synovial Fibroblasts in Rheumatoid Arthritis
Source: Int J Rheum Dis. 2026 Jun 5;29(6):e70690. doi: 10.1111/1756-185x.70690 (PMC13238299; doi:10.1111/1756-185x.70690)
Supplement: Supplementary file 1 — Figure S1: The gating strategy of RASF subsets. Figure S2: siRNA knockdown efficiencies. Figure S3: scRNA‐seq Heatmap. Figure S4: Full cell‐cycle distribution of bulk RASFs treated with GANT61 and TNF‐α. Figure S5: Negative and positive control results for Annexin V/PI apoptosis analysis in bulk RASFs. Figure S6: Uncropped Western blot images for GLI3 detection in freshly isolated RASF subsets. [file APL-29-e70690-s003.pptx]

## Slide 1
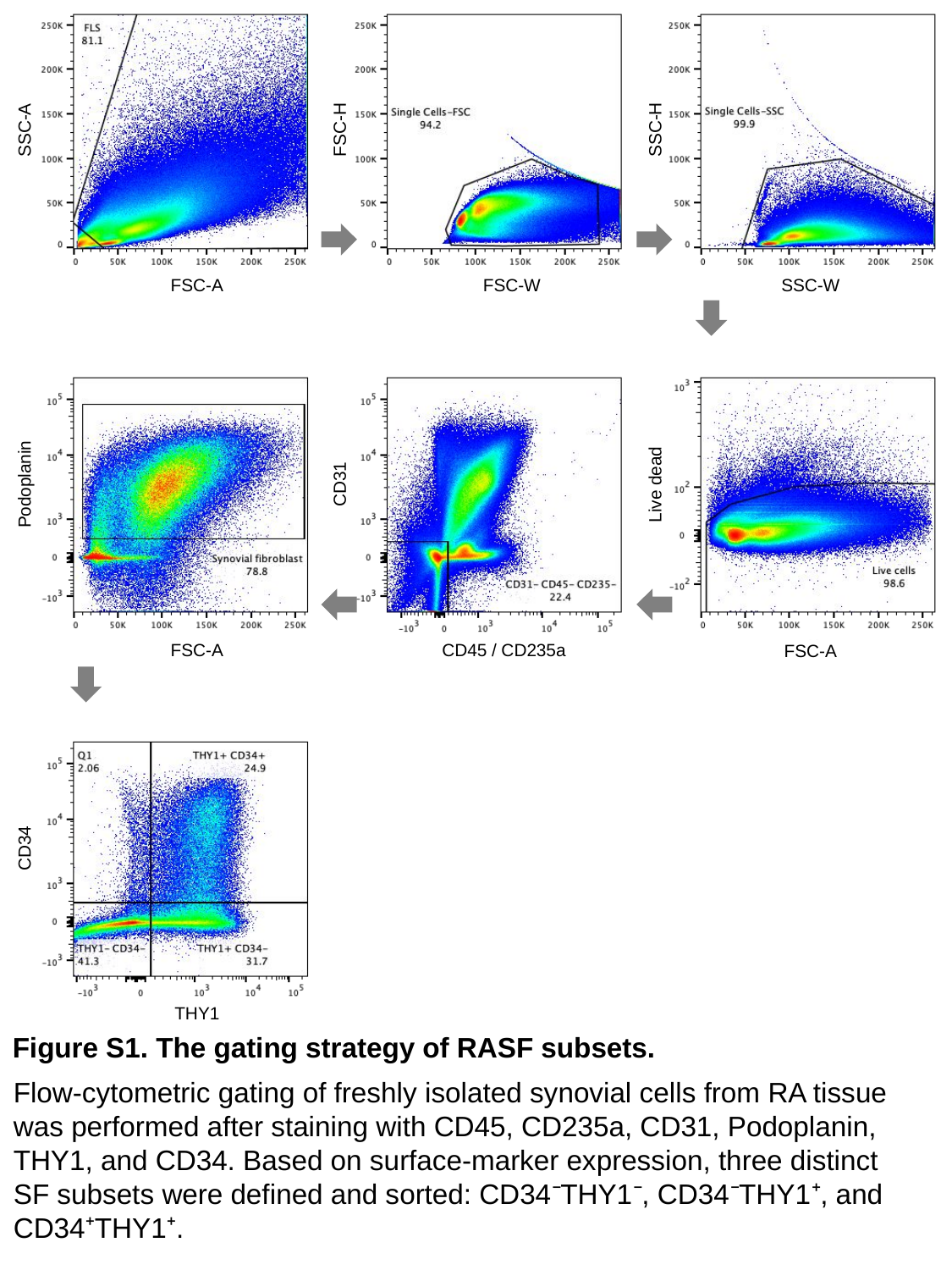

FSC-H
SSC-H
SSC-A
FSC-A
FSC-W
SSC-W
Podoplanin
CD31
Live dead
FSC-A
CD45 / CD235a
FSC-A
CD34
THY1
Figure S1. The gating strategy of RASF subsets.
Flow-cytometric gating of freshly isolated synovial cells from RA tissue was performed after staining with CD45, CD235a, CD31, Podoplanin, THY1, and CD34. Based on surface-marker expression, three distinct SF subsets were defined and sorted: CD34⁻THY1⁻, CD34⁻THY1⁺, and CD34⁺THY1⁺.

## Slide 2
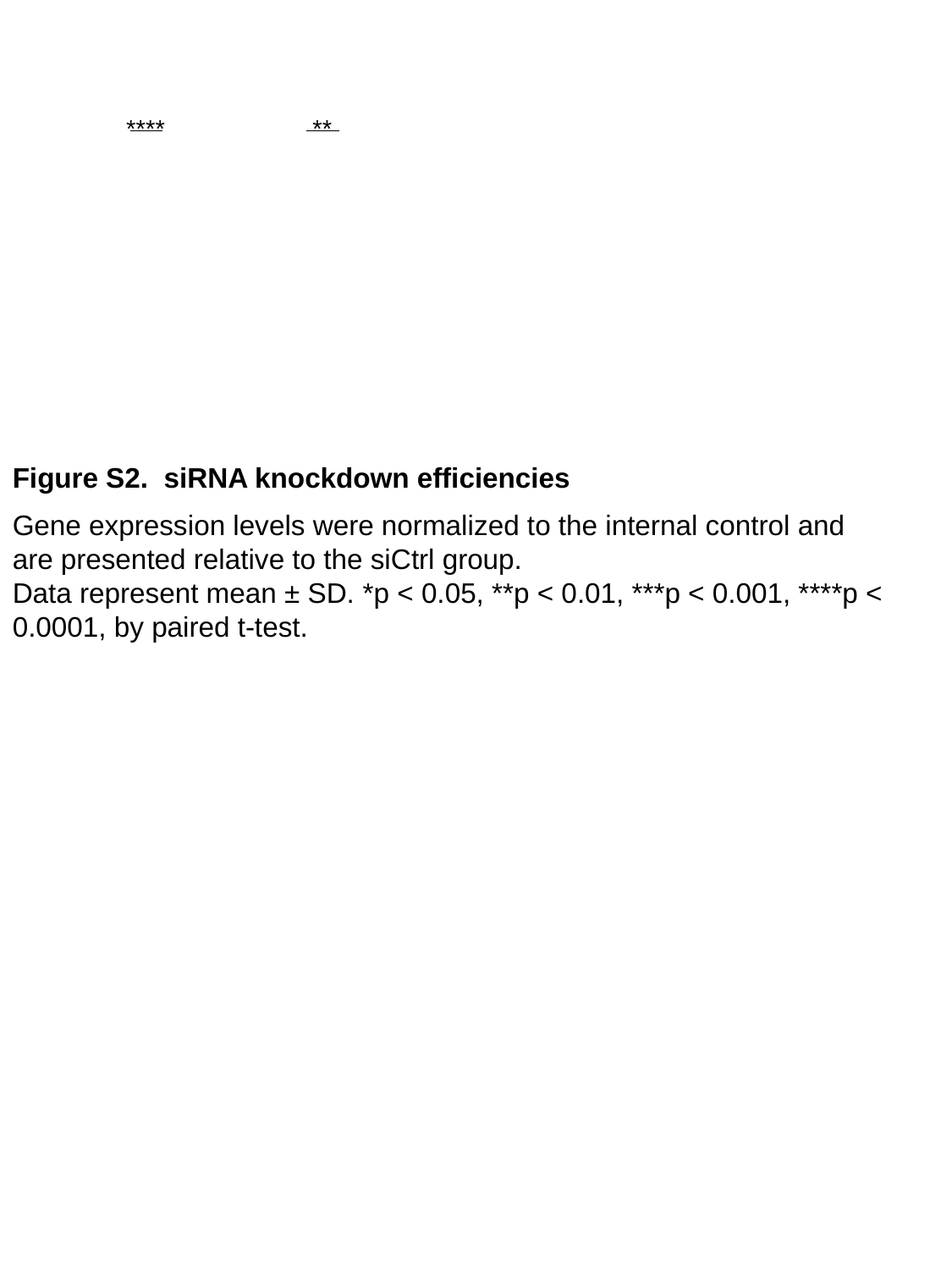

****
**
Figure S2. siRNA knockdown efficiencies
Gene expression levels were normalized to the internal control and are presented relative to the siCtrl group.
Data represent mean ± SD. *p < 0.05, **p < 0.01, ***p < 0.001, ****p < 0.0001, by paired t-test.

## Slide 3
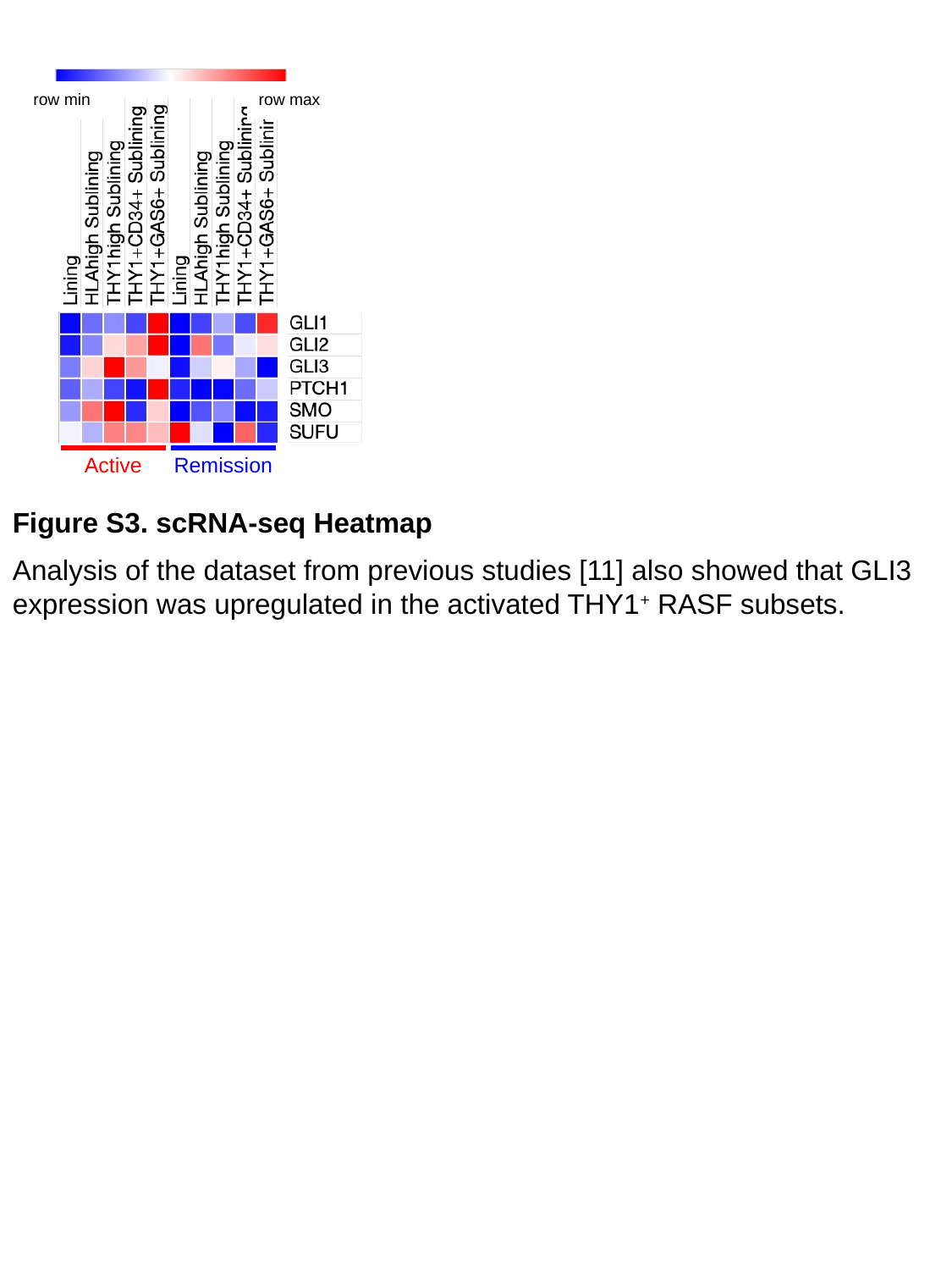

row min
row max
Active
Remission
Figure S3. scRNA-seq Heatmap
Analysis of the dataset from previous studies [11] also showed that GLI3 expression was upregulated in the activated THY1+ RASF subsets.

## Slide 4
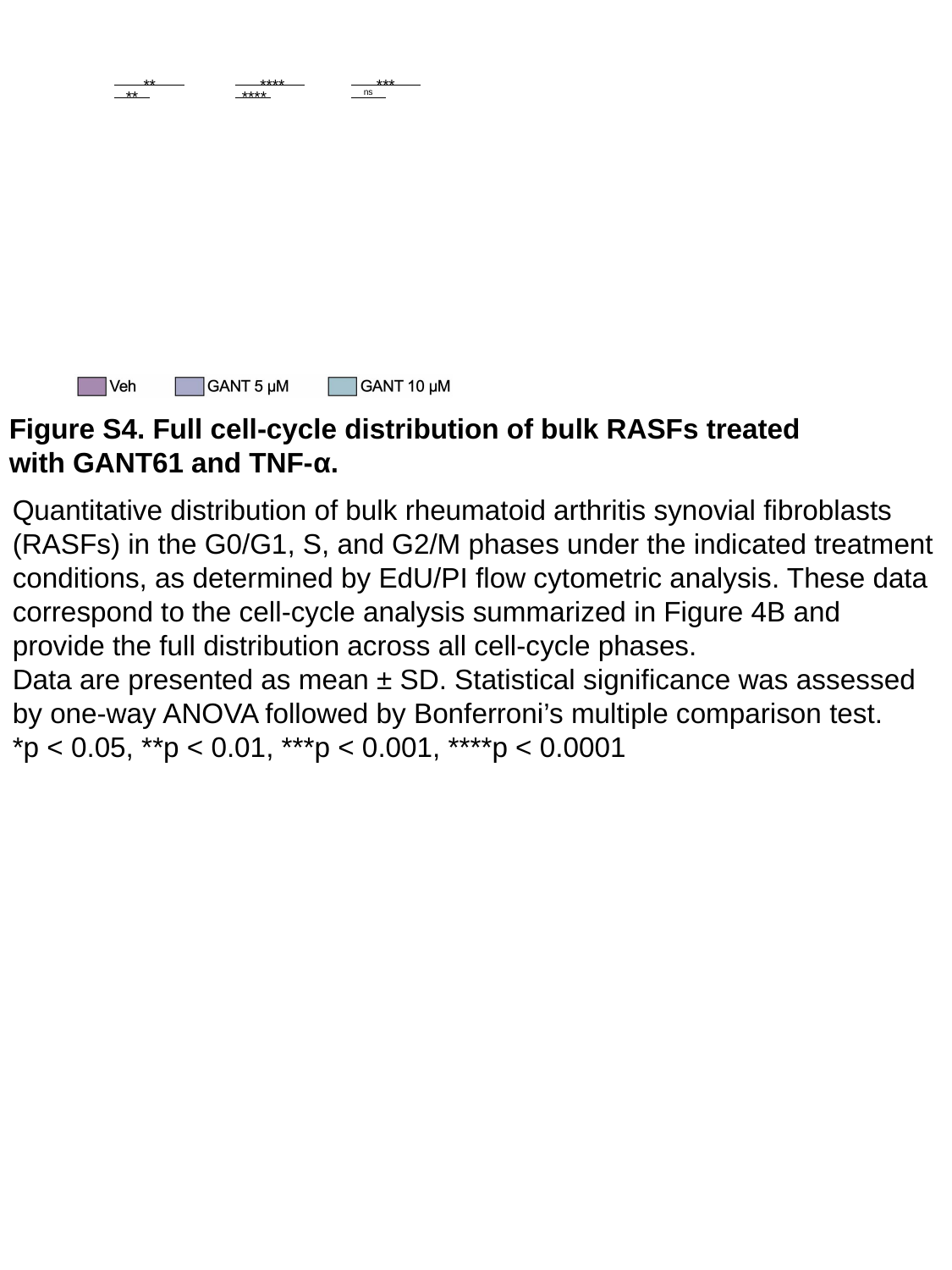

**
****
***
**
****
ns
Figure S4. Full cell-cycle distribution of bulk RASFs treated with GANT61 and TNF-α.
Quantitative distribution of bulk rheumatoid arthritis synovial fibroblasts (RASFs) in the G0/G1, S, and G2/M phases under the indicated treatment conditions, as determined by EdU/PI flow cytometric analysis. These data correspond to the cell-cycle analysis summarized in Figure 4B and provide the full distribution across all cell-cycle phases.
Data are presented as mean ± SD. Statistical significance was assessed by one-way ANOVA followed by Bonferroni’s multiple comparison test.
*p < 0.05, **p < 0.01, ***p < 0.001, ****p < 0.0001

## Slide 5
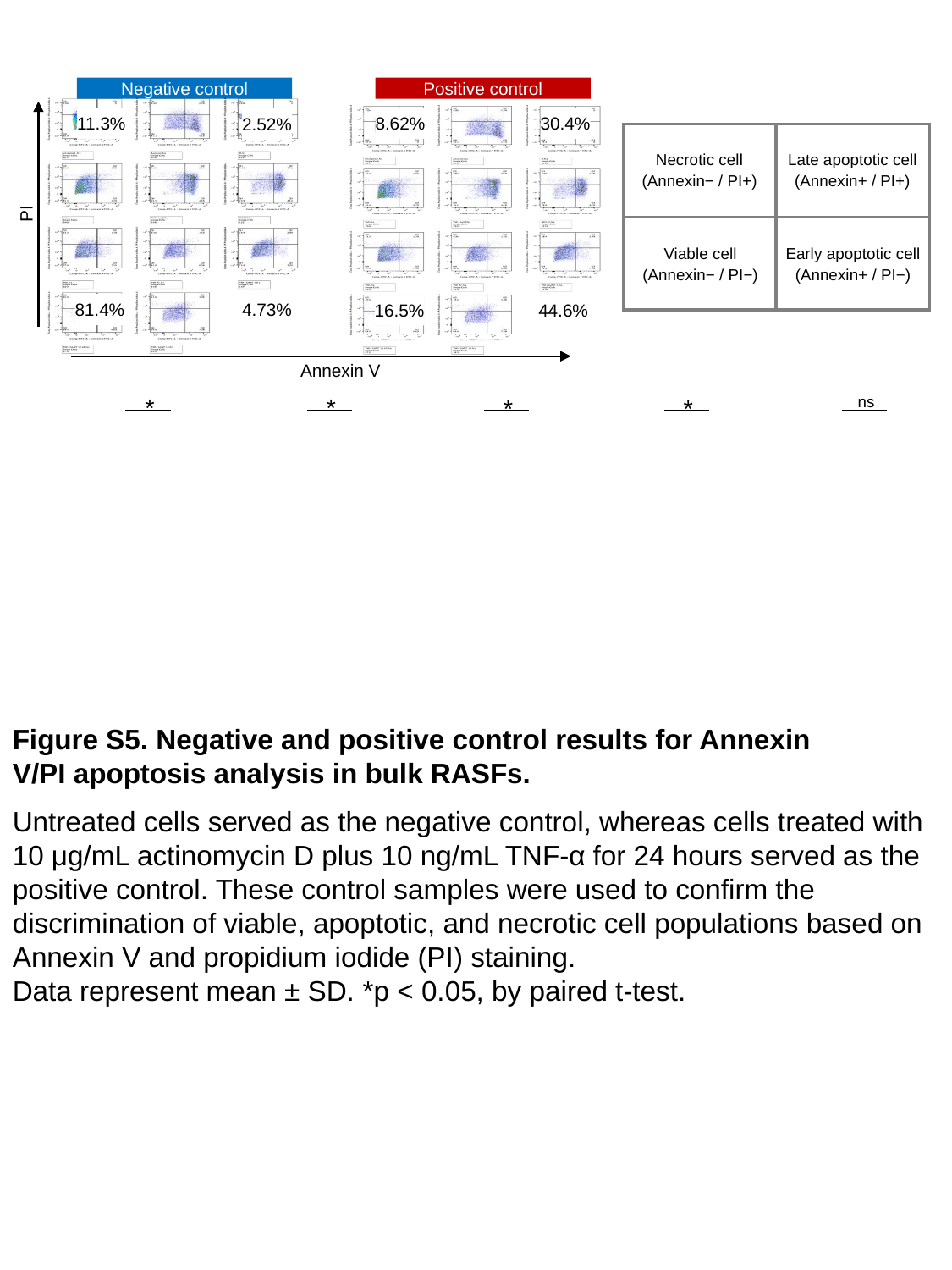

Negative control
Positive control
11.3%
8.62%
30.4%
2.52%
Necrotic cell
(Annexin− / PI+)
Late apoptotic cell
(Annexin+ / PI+)
PI
Viable cell
(Annexin− / PI−)
Early apoptotic cell
(Annexin+ / PI−)
4.73%
81.4%
16.5%
44.6%
Annexin V
ns
*
*
*
*
Figure S5. Negative and positive control results for Annexin V/PI apoptosis analysis in bulk RASFs.
Untreated cells served as the negative control, whereas cells treated with 10 μg/mL actinomycin D plus 10 ng/mL TNF-α for 24 hours served as the positive control. These control samples were used to confirm the discrimination of viable, apoptotic, and necrotic cell populations based on Annexin V and propidium iodide (PI) staining.
Data represent mean ± SD. *p < 0.05, by paired t-test.

## Slide 6
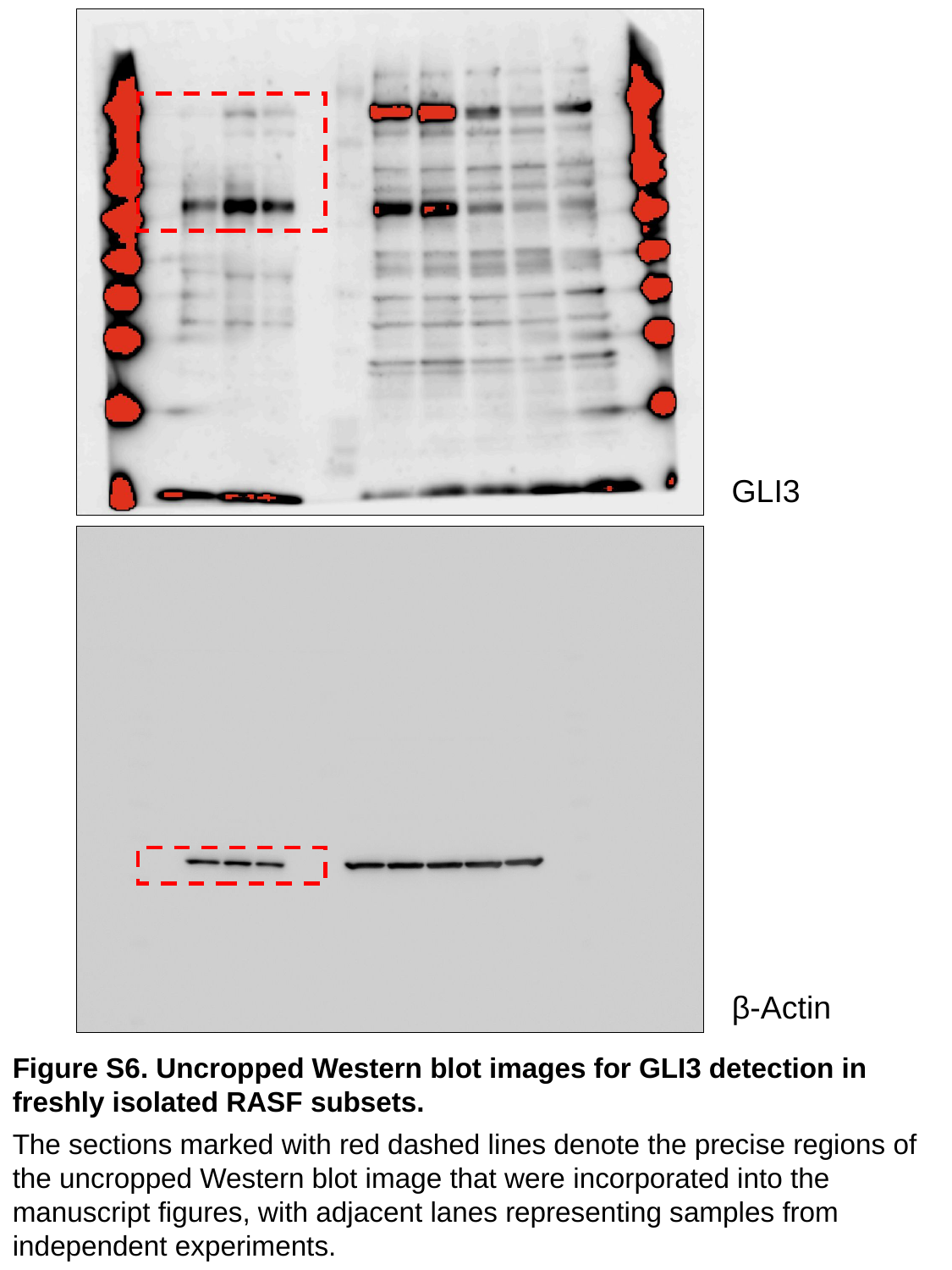

GLI3
β-Actin
Figure S6. Uncropped Western blot images for GLI3 detection in freshly isolated RASF subsets.
The sections marked with red dashed lines denote the precise regions of the uncropped Western blot image that were incorporated into the manuscript figures, with adjacent lanes representing samples from independent experiments.
