# Supplementary material for: Reciprocal Regulation of GLI1 and GLI3 Fine Tunes the Pathogenic Behavior of Synovial Fibroblasts in Rheumatoid Arthritis
Source: Int J Rheum Dis. 2026 Jun 5;29(6):e70690. doi: 10.1111/1756-185x.70690 (PMC13238299; doi:10.1111/1756-185x.70690)
Supplement: Supplementary file 3 — Table S2: qPCR primer sequences. [file APL-29-e70690-s002.docx]

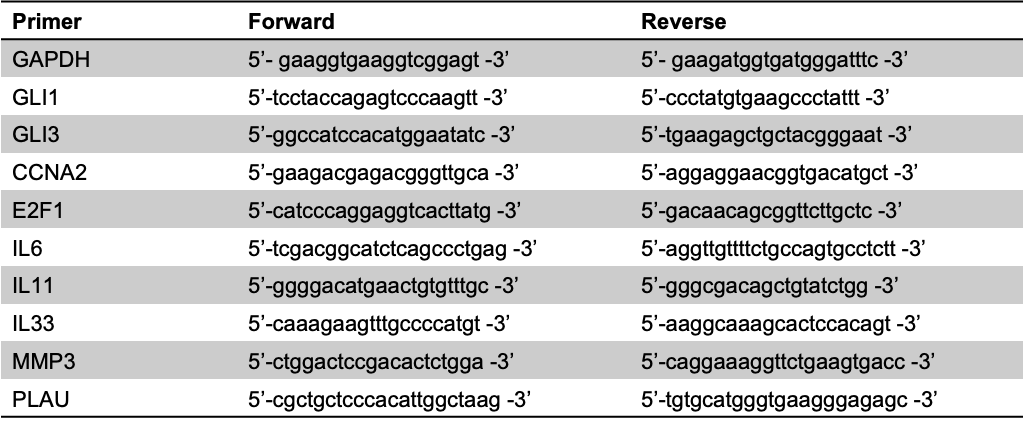


**Table S2. qPCR primer sequences**

List of primer sequences used for qPCR analysis in this study. Gene symbols, forward primer sequences, and reverse primer sequences are shown.
